# Recognition of Emotions From Facial Point-Light Displays

**DOI:** 10.3389/fpsyg.2020.01062

**Published:** 2020-06-04

**Authors:** Christel Bidet-Ildei, Arnaud Decatoire, Sandrine Gil

**Affiliations:** ^1^Université de Poitiers, Poitiers, France; ^2^Université de Tours, Tours, France; ^3^Centre de Recherches sur la Cognition et l’Apprentissage, UMR 7295, Poitiers, France; ^4^Centre National de la Recherche Scientifique (CNRS), Paris, France; ^5^Institut Pprime UPR 3346, Poitiers, France

**Keywords:** emotion, point-light display, face, priming, emotional recognition

## Abstract

Facial emotion recognition occupies a prominent place in emotion psychology. How perceivers recognize messages conveyed by faces can be studied in either an explicit or an implicit way, and using different kinds of facial stimuli. In the present study, we explored for the first time how facial point-light displays (PLDs) (i.e., biological motion with minimal perceptual properties) can elicit both explicit and implicit mechanisms of facial emotion recognition. Participants completed tasks of explicit or implicit facial emotion recognition from PLDs. Results showed that point-light stimuli are sufficient to allow facial emotion recognition, be it explicit and implicit. We argue that this finding could encourage the use of PLDs in research on the perception of emotional cues from faces.

## Introduction

Presumably because of their crucial role in everyday life, facial expressions have become an important research topic in the field of human interactions. They are key to social communication, as they can promote mental state inference and emotion induction in the perceiver (e.g., Wood et al., 2016). In this context, research on point-light displays (PLDs) imitating biological motion has expanded considerably. The aim of the present study was to investigate whether facial PLDs can elicit two important processes: explicit (explicit judgment) and implicit (via a priming task) emotion recognition.

Researchers study facial emotion recognition using either explicit or implicit tasks. In the former, which are widely used, participants have to explicitly judge emotions conveyed by target faces (e.g., Calvo and Lundqvist, 2008; Adolph and Alpers, 2010). In the latter, they perform a priming task, where instead of responding about a face representing the prime stimulus, they respond to a probe stimulus following the presentation of the prime (e.g., McLellan et al., 2010; Wentura et al., 2017). Here, the congruency effect implies that the probe stimulus judgment is influenced by the emotional information conveyed by the prime. For instance, a probe stimulus is judged to be more negative, and the reaction time is shorter, when it is preceded by a negative rather than a positive prime. Implicit perception is thought to rely on an early stage of processing (i.e., automatic mechanisms) that is nonconscious and functional even with poor stimuli. By contrast, explicit recognition involves the linking of raw perceptions to relevant conceptual knowledge and inference processes (Adolphs, 2002). Neuroscience studies (see Dricu and Frühholz, 2016, for a meta-analysis) and behavioral findings in individuals with psychiatric disorders (e.g., Wagenbreth et al., 2016) argue in favor of this distinction.

Another issue in the field of emotional faces concerns the properties of the stimuli that are used. As summarized in a recent review (Dobs et al., 2018), different types of facial stimuli can be considered (e.g., static vs dynamic; real vs synthetic). Although most research has looked at static faces, a number of studies have suggested that dynamic facial expressions benefit recognition in certain conditions (for a review, see Krumhuber et al., 2013), even if interpretations (e.g., attention driven, facilitation of mimicry behavior) are still sparse (e.g., Calvo et al., 2016). Given their respective advantages and disadvantages (Dobs et al., 2018), stimuli need to be chosen according to the research objective. In this vein, studies featuring PLDs are growing in popularity.

A PLD is a stylised depiction of the articulated motion (i.e., intrinsic properties) of a living creature (human or other animal) (Troje and Aust, 2013). PLDs can be useful in social research, as they provide a way of studying core social cues, while controlling other perceptual dimensions (e.g., color, image attractiveness). Since the introduction of the point-light motion methodology in the early 1970s (Johansson, 1973), numerous studies have shown that humans are very sensitive to kinematic information. Simply by watching moving dots representing an actor performing an action, people can recognize the action being produced (Johansson, 1973), or access different features of the actor, such as the sex, intention, or identity (see Decatoire et al., 2018, for a review). Importantly, this ability may be compromised in patients with social cognition disorders such as autism (e.g., Blake et al., 2003) or schizophrenia (e.g., Kim et al., 2005), suggesting that visual body motion processing is a hallmark of social cognition (Pavlova, 2012). Consequently, some authors have used PLDs to study the recognition of emotional states (e.g., Atkinson et al., 2004; Alaerts et al., 2011), and have shown that emotion can be accurately recognized simply from watching a moving body. In a recent review, Okruszek (2018) summarized the performances of patients with different disorders on emotion recognition from body PLDs. He noted that recognition of emotion from body PLDs seems to be specifically affected in patients, suggesting that this is a relevant methodology for studying social problems in patients with psychiatric disorders.

Although body PLDs are now used to assess the recognition of emotions, to our knowledge, very few studies have specifically investigated the ability to recognize emotions from facial PLDs (Bassili, 1978, 1979; Dittrich, 1991; Pollick et al., 2003; Atkinson et al., 2012). In these studies, the authors showed that adults are able to recognize basic emotional expressions from dynamic stimuli. Crucially, these studies only investigated explicit emotion recognition.

The aim of the present study was to assess whether emotional facial PLD stimuli can elicit both explicit and implicit facial emotion recognition processes. In addition, we explored the possible effect of sex, as some studies have reported a *female advantage* both for emotional faces (Hall et al., 2000; Fischer et al., 2018) and for threatening biological-motion stimuli (Alaerts et al., 2011; Pavlova et al., 2015). In the present study, participants performed two kinds of task: an explicit emotional facial PLD recognition task (open-ended and multiple-choice questionnaires), and a priming paradigm with PLDs as the primes. We expected to observe good performance on the explicit emotion recognition tasks, similar to the rates of recognition usually observed in the literature for full-light emotional faces (Nelson and Russell, 2013) and PLD faces (Bassili, 1979), and a congruency effect in the implicit task, with both effects being potentially modulated by the perceiver’s sex.”

## Methods

### Participants

Participants were thirty-seven French university students^1^ (*M*_*age*_ = 20.9 years, *SD* = 1.61; 17 male; nine left-handed). They all provided their written informed consent in accordance with the Declaration of Helsinski, and took part in exchange for a course credit. All participants had normal or corrected-to-normal vision, and no history of motor, perceptual or neurological disorders, as assessed with a short questionnaire. The protocol was approved by a local human subject committee.

### Material

Eight PLDs depicting an emotional facial expression (happiness, anger, disgust, or surprise) of a man or woman were used in the experiment. These PLDs were taken from the PLAViMoP database (Decatoire et al., 2018; https://plavimop.prd.fr/en/motions^1^). They were each made up of 41 points representing the position of the actors’ eyes, eyebrows, mouth, nose, outline of their face, shoulders and sternum. 4 mm hemispherical facial markers were used for face and 6.4 mm spherical markers for sternum and shoulders (see Appendix 1 and Image 1 in Supplementary Material for a complete description). Importantly, the eyes’ position has not been recorded directly but has been calculated a posteriori from the position of dots placed at the edge of each eye. This choice was made to render our PLDs more “face like” because a pre-test including 15 adult participants showed that without eyes, our face PLDs seemed strange and ghostly. We extracted 60 neutral nouns composed of one (30 nouns) or two (30 nouns) syllables from the validated French Affective Norms database (Monnier and Syssau, 2014). These French words were selected for their neutral valence (*M* = 4.88, *SD* = 1.53) and level of arousal (*M* = 3.30, *SD* = 2.22), as rated on the nine-point scale of the database.

### Procedure

The participants were tested individually in a dark, soundproof room, seated in front of a computer screen (spatial resolution: 1280 × 800 pixels; temporal resolution: 60 Hz). Three tasks were administered in the following order: priming task (implicit emotion evaluation), and the open-ended and multiple-choice questionnaires (explicit emotion evaluation) for a total duration of 45 min.

#### Implicit Emotion Evaluation: Priming Task

**FIGURE 1:**
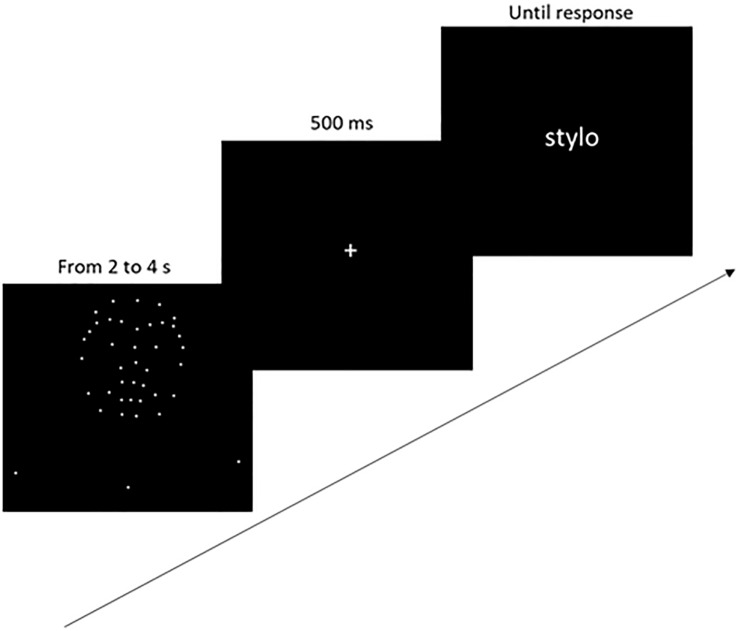
Priming task procedure. The point-light sequence, fixation cross, and word stimulus were successively displayed in the center of the screen. The arrow represents the sequence of one trial.

The experimental session featured 240 random trials (four PLDs × 60 words). The PLDs used in this session were male anger, female anger, male happiness and female happiness. Each trial consisted in the presentation of a PLD (lasting 2-4 s) followed by a fixation cross (500 ms), then a neutral word (see Figure 1). The word remained on the screen until the participant entered a response. The participants’ task was to judge, as quickly and as accurately as possible, whether the word presented was positive or negative. Participants answered by pressing the P or A keys of an AZERTY keyboard with their right or left hand. The keys associated with the positive and negative responses were counterbalanced between participants.

Before the experimental session, we administered a training session using the other PLD sequences (male surprise, male disgust, female surprise, female disgust) and different target words.

#### Explicit Emotion Evaluation: Open-Ended and Multiple-Choice Questionnaires

In the open-ended questionnaire, the PLDs that had been used in either the training or the experimental session were presented in random order, and participants were asked, “What is it?” after each one. No PLD was presented more than twice. We recorded the participants’ responses for each PLD. No limit time was given, but the experimenter encouraged participants to be spontaneous.

In the multiple-choice questionnaire, the previously used PLDs were again presented to participants in random order. For each one, participants had to judge which of the four emotions (anger, happiness, disgust or surprise) was featured. They also had to rate the intensity of the PLD on a scale ranging from 1 (*Very low*) to 7 (*Very high*) and its valence on a scale ranging from 1 (*Very negative*) to 7 (*Very positive*). We recorded the participants’ responses for each PLD. No limit time was given, but the experimenter encouraged participants to be spontaneous.

### Data Analysis

In the priming task, our first dependent variable (DV) was participants’ binary responses to categorizing neutral words (i.e., *negative* vs *positive*) as a function of PLD prime (i.e., negative or positive expression). Each response was coded one when it was congruent with the valence of the PLD prime (i.e., *positive* response when the PLD represented a positive expression; *negative* response when the PLD represented a negative expression), and 0 when it was incongruent. The second DV was the corresponding response times. For these two DVs, we calculated mixed models, using the GLIMMIX procedure with SAS Version 9.4 statistical software. For the first DV (i.e., categorisation), we ran a logistic mixed model, with participants and items as random-effects factors. Three fixed-effects factors were considered: PLD category (positive/happiness vs negative/anger), sex of participants (male or female), and their interactions (see Baayen et al., 2008). For the second DV (i.e., reaction times), the analysis used a gamma distribution as recommended (Baayen and Milin, 2010). The same three fixed-effects factors and their interactions were considered. For all analyses, we calculated the *p* values for the reported *F* values (Type III analysis of variance, ANOVA) with the error degrees of freedom calculated according to the Satterthwaite approximation, as the number of observations varied across conditions. The significance level was set at *p* = 0.05.

For the open-ended questionnaire and the multiple-choice questionnaire, the scores were compared using a nonparametric Friedman ANOVA. The difference between males and females was then assessed with Mann-Whitney comparisons. Moreover, for the multiple-choice questionnaire, we compared the mean percentage of correct emotion recognition responses with chance level (25%) using a *z* test. The data that support the findings of this study are openly available in figshare at http://doi.org/10.6084/m9.figshare.11407611.

## Results

### Implicit Emotion Evaluation: Priming Task

**FIGURE 2:**
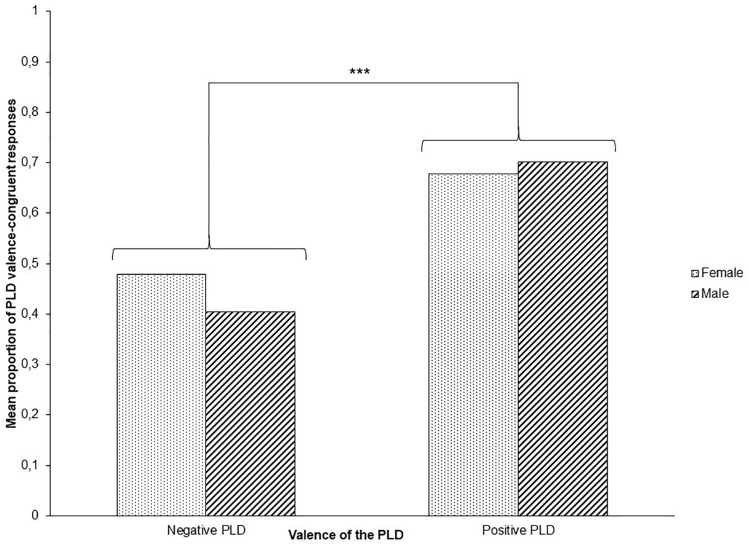
Mean proportion of congruent responses as a function of prime (PLD expression) valence (i.e., negative vs positive). The asterisks indicate a significance level of *p* < 0.001.

The logistic statistical model revealed that the nature of the PLD was predictive of congruent responses when these were positive (see Figure 2). Participants categorized neutral words as being more positive when they were primed by a positive rather than a negative PLD, *F*(1,128) = 40.47, *p* < 0.0001, 95% CI = [0.173, 0.397]. No other effect was significant (all *p*s > 0.1).

**FIGURE 3:**
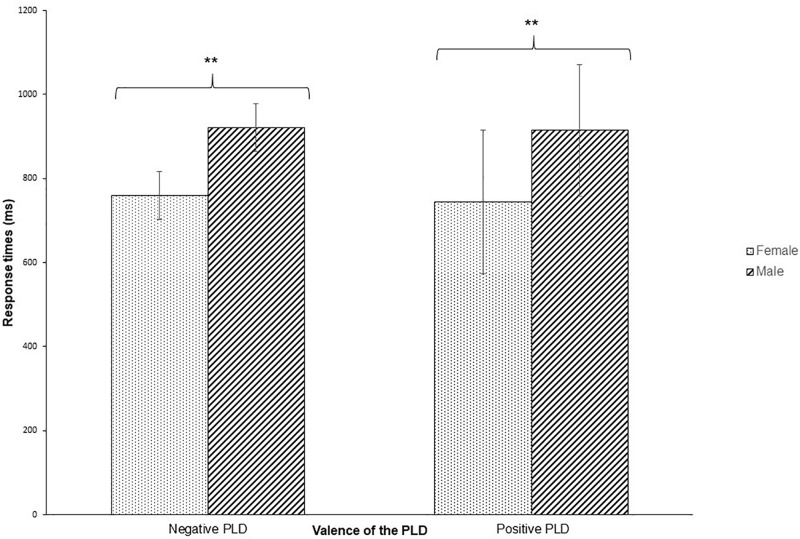
Mean response times as a function of prime (PLD expression) valence (i.e., negative vs positive). Error bars represent 95% confidence intervals. The asterisks indicate a significance level of *p* < 0.01.

Concerning response times (see Figure 3), analysis showed an effect of sex of participants, *F*(1,145.8) = 8.86 *p* = 0.003, with faster responses for females (*M* = 752.3 ms, SD = 125.7 ms) than for males (*M* = 917.5 ms, SD = 313.6 ms). No other main or interaction effect was significant (all *p*s > 0.17).

### Explicit Emotion Evaluation: Open-Ended and the Multiple-Choice Questionnaires

Concerning responses to the open-ended questionnaire, analysis revealed an effect of emotion, chi^2^(*N* = 37, *df* = 3) = 63.68, *p* < 0.001, with better recognition for happiness (*M* = 90.5%, SD = 19.8%) and surprise (*M* = 93.2%, SD = 20.9%) than for anger (*M* = 56.8%, SD = 26.8%) and disgust (*M* = 37.8%, SD = 36.1%). There was no difference between males and females on the percentage of correct emotion recognition responses (all *p*s > 0.32).

For the multiple-choice questionnaire, results showed an effect of emotion, chi^2^(*N* = 37, *df* = 3) = 34.26, *p* < 0.001, with better recognition for surprise (*M* = 100%) and happiness (*M* = 97.2%, SD = 11.4%) than for disgust (*M* = 81%, SD = 27.2%) and anger (*M* = 75.7%, SD = 27.9%). There was no difference between males and females on the percentage of correct emotion recognition responses (all *p*s > 0.55), and the recognition score was above chance level for all PLDs (all *p*s < 0.001).

Concerning intensity ratings, there was a significant effect of emotion, chi^2^(*N* = 37, *df* = 3) = 53.27, *p* < 0.001, with higher ratings for happiness (*M* = 5.87, SD = 0.58) than for either anger (*M* = 4.55, SD = 0.90), surprise (*M* = 4.06, SD = 1.47), or disgust (*M* = 3.59, SD = 1.08). There was no difference between males and females on judgments of intensity (all *p*s > 0.12).

Concerning valence ratings, we observed a significant effect of emotion, chi^2^(*N* = 37, *df* = 3) = 86.33, *p* < 0.001, with higher ratings for happiness (*M* = 5.43, SD = 0.71) and surprise (*M* = 3.97, SD = 0.78) than for disgust (*M* = 3.01, SD = 0.58) and anger (*M* = 2.45, SD = 0.63). There was no difference between males and females except on surprise (*U* = 97, *p* < 0.05), to which males (*M* = 4.32, *SD* = 0.64) attributed a higher valence than females did (*M* = 3.68, *SD* = 0.78).

## Discussion

The aim of the present study was to assess whether the presentation of emotional facial PLDs can elicit both explicit and implicit processes of facial emotion recognition. Concerning explicit emotion recognition, results showed similar performance that these observed in literature for full-light emotions and comparable methodology (for multiple-force choice tasks, see for example the review’s of Nelson and Russell (2013) based on 39 sets of data) and PLD emotions (Bassili, 1979). Moreover, our performances are above chance level for all four emotions proposed which suggests that our stimuli are adequate to assess emotions. However, this result should be confirmed in futures studies in particular to assess how our calculation of the eyes’ position can affect the recognition. Actually, our calculation gives all the stimuli the impression that the actors are looking at the camera and this is maybe not so natural for some emotions such as disgust for example. Moreover, several works have shown that the recognition of PLD faces are dependant of spatial and dynamic manipulations (e.g., Pollick et al., 2003) and that the modification of stimuli can affect the cerebral areas activated (Atkinson et al., 2012). Future studies should assess this point with stimuli associating classical PLD recording and pupil recording. Interestingly, we obtained better recognition of happiness and surprise than of anger and disgust. This difference was observed whether the recognition was open-ended or constrained by multiple choices. Our findings confirm those reported by previous studies using facial PLDs (e.g., Bassili, 1979; Pollick et al., 2003; Atkinson et al., 2012). More importantly, both the high explicit emotion recognition scores and the hierarchy between the different valences (i.e., positive emotions recognized better than negative emotions) were consistent with previous findings for full-light facial stimuli (for a review, see Nelson and Russell, 2013). As with full-light facial expressions, the easier recognition of positive facial PLDs, generally called the *happiness advantage* in the literature (e.g., Leppänen and Hietanen, 2004), can be explained by different perceptual or theoretical features/functions of positive faces (e.g., mouth region processing; Sullivan et al., 2007; Calvo and Beltrán, 2013). Globally, the explicit task findings suggest that facial PLDs are recognized largely above chance level (for the multiple choice task) and are just as efficient as full-light facial expressions (Nelson and Russell, 2013). Even these results could be confirmed with a direct comparison group control, they suggest that PLD faces are good stimuli for expressing emotions.

Concerning the implicit recognition of emotional facial PLDs, our findings show that PLDs are sufficient to trigger a congruency effect. When participants saw a positive facial PLD as a prime, they had a significant tendency to attribute a positive valence to subsequent neutral words. However, this effect was not observed for negative emotions. This discrepancy can be explained either by a lower level of recognition of negative emotions or by a specificity of positive emotions, as explained in the previous section dealing with explicit recognition.

Concerning the effect of sex, the analysis of response times indicated that women were more efficient than men in judging emotional facial PLDs in the implicit task. This could be related to the known *f. advantage* observed for both emotional faces (Hall et al., 2000; Kret and De Gelder, 2012; Fischer et al., 2018) and biological-motion stimuli (Alaerts et al., 2011; Pavlova et al., 2015), as mentioned in the Introduction. However, as this advantage was only observed on response times, we can hypothesize that it is related to two kinds of sex differences. First, the literature highlights females’ ability to respond more automatically than males to minimal affective stimuli, as in subliminal presentations (e.g., Hall and Matsumoto, 2004; Hoffmann et al., 2010; Donges et al., 2012). Second, several studies have pointed to sex differences in the time course and topography of the neural circuitry underpinning biological motion processing (Pavlova et al., 2015). Therefore, we suggest that sex differences in the analysis of facial PLDs are related to the automatic capture of women’s attention (Bidet-Ildei and Bouquet, 2014).

More generally speaking, our main finding was to show that a minimalistic representation of emotional faces like PLDs seems to convey critical visual information for expression recognition. This result is consistent with the literature showing that humans are skilled at processing different kinds of social information (e.g., gender, intention, affective state) with minimalistic representation (see Troje and Aust, 2013 for a review). Moreover, the present study expands on this literature by suggesting that this efficient treatment is also the case on the basis of emotional facial PLDs. One interpretation is offered by the framework of embodied cognition (Barsalou, 1999, 2010; Niedenthal, 2007), which argues that the processing of emotional stimuli can be supported by somatosensory reactions. PLDs – even if they are minimalistic stimuli – are by definition based on “Life motion” (i.e., they correspond to the biological dynamic of human actions). Therefore, emotional PLDs can be considered as a privileged way for embodied emotional recognition responses. Precisely, in simulation theories, the internal simulation of movements is a prominent mechanism of efficient emotion recognition; a growing literature in neuroimaging studies in both typical and atypical populations argues for emotion recognition reliance to somatosensory cortices (for a recent review, see Ross and Atkinson, 2020).

Finally, emotional facial PLDs can be employed to efficiently assess both explicit and implicit processes of facial emotion recognition. Therefore, they could serve as socio-emotional cues instead of videos and static materials, especially when researchers wish to control and/or limit the amount of information available. Facial PLDs can provide an easy manner to study the dynamics and the number of clues available during a facial emotion recognition task (Decatoire et al., 2019). For instance, they offer the opportunity to examine the role of biological dynamic in emotion recognition (for example, see Atkinson et al., 2007, using upright or inverted PLDs of body). In the same manner, they could be applied to examine the minimum amount of information required for the recognition of a given emotion (e.g., by changing the color or the size of some dots) (see also Pollick et al., 2003 for spatial and dynamic manipulations of PLDs). Moreover, facial PLDs could be a useful tool to study emotion recognition mechanisms in some patients (e.g., Okruszek, 2018), such as people with autism spectrum disorder (for a review, see Tanaka and Sung, 2016). In conclusion, emotional facial PLDs could constitute both a theoretical and a practical interest to better understand the mechanisms involved in the recognition of emotions.

## Data Availability Statement

The datasets generated for this study are openly available in figshare at http://doi.org/10.6084/m9.figshare.11407611.

## Ethics Statement

Ethical review and approval was not required for the study on human participants in accordance with the local legislation and institutional requirements. The patients/participants provided their written informed consent to participate in this study.

## Author Contributions

CB-I and SG both conceptualized the study and did analysis of the data. AD participated to the building of the stimuli. CB-I constructed the software programs, conducted and supervised data collection. CB-I and SG both contributed to writing of this manuscript.

## Conflict of Interest

The authors declare that the research was conducted in the absence of any commercial or financial relationships that could be construed as a potential conflict of interest.

## Footnotes

1 Given than we anticipated to use logistic mixed models for our analysis (see data analysis), we did not estimate the *a priori* sample size which is not adapted for this type of analysis.

## Supplementary Material

FIGURE S1 Placement of the markers used to make the PLDs used in the experiment.

VIDEO S1 Female disgust.

VIDEO S2 Male disgust.

VIDEO S3 Female joy.

VIDEO S4 Male joy.

VIDEO S5 Female surprise.

VIDEO S6 Male surprise.

VIDEO S7 Female anger.

VIDEO S8 Male anger.

## Appendix 1

Placement of the markers used to make the PLDs used in the experiment. For more information, please consult https://plavimop.prd.fr/en/motions.

38 markers in the face +3 (Shoulders and plexus)

8 Eyebrows: REB1/REB2/REB3/REB4/LEB1/LEB2/LEB3/LEB42 Eyes: ER/EL

5 Nose: N1/N2/N3/NL/NR

8 Mouth: M1/M2/M3/M4/M5/M6/M7/M8

15 Face: F1/F2/F3/F4/F5/F6/F7/F8/F9/F10/F11/F12/F13/F14/F15

+ RSHO/LSHO/STER. See also Image 1 in Supplementary Material.
