# Supplementary figures and images for: Recognition of Emotions From Facial Point-Light Displays
Source: Front Psychol. 2020 Jun 4;11:1062. doi: 10.3389/fpsyg.2020.01062 (PMC7287185; doi:10.3389/fpsyg.2020.01062)

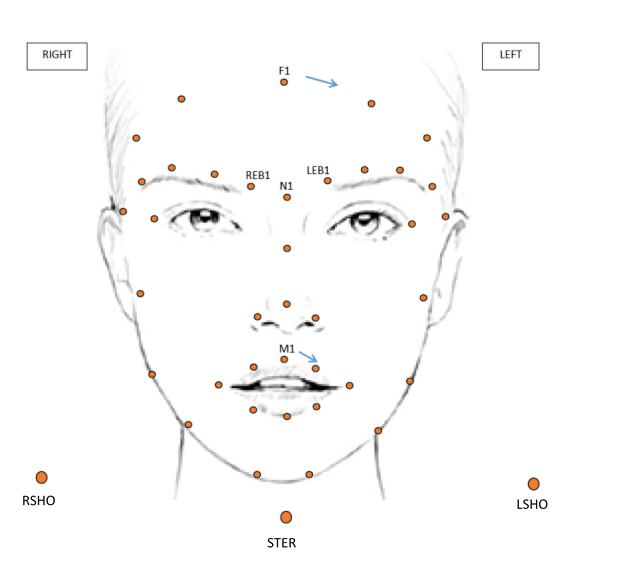

Supplement: FIGURE S1 — Placement of the markers used to make the PLDs used in the experiment. [file Image_1.TIF]

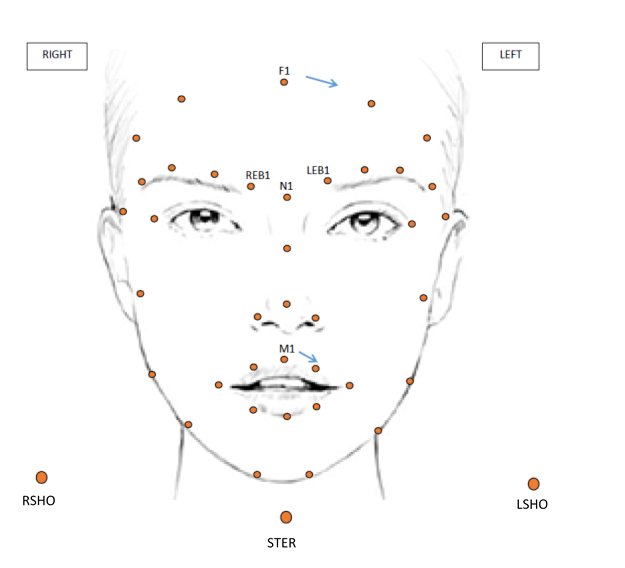


Right Reference

Marker (R)

Left Reference

Marker (L)

Right Eye (E1)

Left Eye (E2)

O

X

Y

With

Supplement: Supplementary file 10 [file Data_Sheet_1.docx]
